# The current image of single SnO_2 _nanobelt nanodevice studied by conductive atomic force microscopy

**DOI:** 10.1186/1556-276X-6-541

**Published:** 2011-10-04

**Authors:** Shujie Wang, Gang Cheng, Ke Cheng, Xiaohong Jiang, Zuliang Du

**Affiliations:** 1 Key Laboratory for Special Functional Materials, Henan University, Kaifeng 475004, People's Republic of China

**Keywords:** SnO_2 _nanobelt, C-AFM, current image

## Abstract

A single SnO_2 _nanobelt was assembled on a pair of Au electrodes by electric-field assembly method. The electronic transport property of single SnO_2 _nanobelt was studied by conductive atomic force microscopy (C-AFM). Back-to-back Schottky barrier-type junctions were created between AFM tip/SnO_2 _nanobelt/Au electrode which can be concluded from the I-V curve. The current images of single SnO_2 _nanobelt nanodevices were also studied by C-AFM techniques, which showed stripes patterns on the nanobelt surface. The current images of the nanobelt devices correlate the microscopy with separate transport properties measurement together.

## Introduction

As an important wide band n-type semiconductor, SnO_2 _possesses many unique optical and electrical properties which have been widely used in optoelectronic devices and gas sensors [1-4]. One dimensional (1-D) SnO_2 _have been reported to have some different characteristics from the bulk crystal due to its large surface-to-volume ratio [5]. Nanodevices based on 1-D SnO_2 _nanostructures have been fabricated and showed significant potential for applications ranging from field-effect transistors, gas sensors, displays, as well as solar cells [6-9]. Although promising results of the gas sensing and other performance of 1-D SnO_2 _have been reported, the development of highly sensitized devices remains a future challenge. Usually, the surface atoms and states on the (1-D) SnO_2 _surface play an important role in its transport behavior which complicates the nanodevice characterization [10]. Recent research result showed that the surface states indeed existed in these wires which could be detected in a contactless manner by spectral analysis [11]. Thus the better understanding of the surface states affected the device transport property needed. The transport property of the nanobelts device and the surface states on the (1-D) SnO_2 _surface must be cared in order to fabricate the practical application of nanodevices. It is difficult for us investigating the transport property of one single nanobelt in the nanometer scale before atomic force microscopy (AFM), especially the conductive AFM (C-AFM) with a conductive tip; in recent years, more and more are used to investigate the transport property and the surface property on the nanostructure in microscope scales [12-15]. AFM tip coated with metal can serve as the conducting electrode, and the transport property of the nanowires can be studied through the I-V curve recorded by C-AFM technique [16]. Furthermore, the current image, simultaneously with the AFM topography, can provide the direct information how the current flows through the nanostructure surface [17,18]. In this paper, singe SnO_2 _nanobelt device is assembled across opposing Au electrodes by electric-field assembly method [19]. Nonlinear and asymmetric I-V curve are obtained by applying a small voltage onto the conductive AFM tip positioned directly on the surface of SnO_2 _nanobelt. We conclude the nonlinear and asymmetric I-V behavior resulted from the back-to-back Schottky barriers between AFM tip/SnO_2 _nanobelt/electrode, and the Schottky barriers is related to the surface states on the nanobelt surface. The current images of the nanobelt device are also studied by C-AFM techniques, which show the current flow through the single nanobelt devices clearly. The results showed that the surface states can affect the transport property of the nanobelt device and display stripe patterns in the current images.

## Experiment

SnO_2 _nanobelts are synthesized using techniques described in Ref. [20]. Briefly, a horizontal alumina tube (outer diameter, 3.7 mm; length, 120 cm) is mounted inside a high-temperature tube furnace. A mixture of SnO_2 _and graphite powders is placed on an alumina wafer. After transferring the wafer to the center of the alumina tube, the tube is evacuated by a mechanical rotary pump to a pressure of 6 × 10^-2 ^Torr. During the experiment, a constant flow of N_2 _is maintained, and the pump continually evacuated the system to keep the pressure inside the tube. The temperature of the furnace is increased to 900°C from room temperature and kept at 900°C for 1 h. After the furnace is cooled down to room temperature, a white wool-like product is formed in a high yield on Si wafer near the boat of the wafer.

In order to fabricate one single SnO_2 _nanobelt nanodevice, the nanobelts are assembled on a pair of Au electrodes by electric-field assembly method [21]. The SnO_2 _nanobelts were ultrasonically dispersed in ethanol, and then the dispersed SnO_2 _nanobelts were deposited on predefined Au electrodes using the electric-field assembly technique. After applying a droplet of the SnO_2 _nanobelt suspension onto the electrodes, the electrodes were connected to a 10 V and 50-kHz AC signal, which was chosen for optimizing the alignment of a single nanobelt. This signal generated an alternating electrostatic force on the nanobelts in the solution. Under the electrical polarization force, the nanobelts were deposited on the electrodes.

## Results and discussion

**Figure 1:**
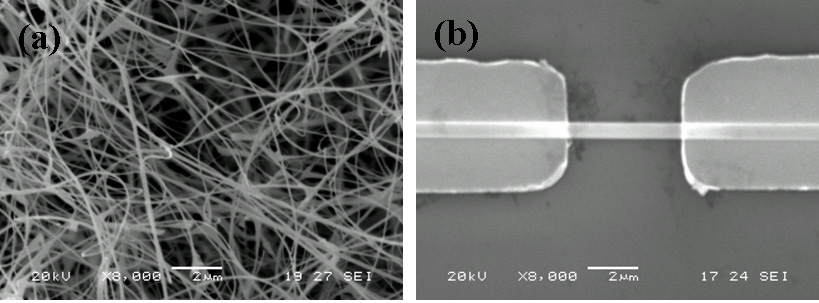
**SEM images of SnO_2 _nanobelt**. (**a**) SEM images of SnO_2 _nanobelt. (**b**) SEM image of a single SnO_2 _nanobelt across the Au electrodes.

A typical scanning electron microscopy (SEM) image of Figure 1a shows that the as-synthesized products consist of nanometer belt-like structures. Figure 1b shows the SEM image of the assembled nanobelt which confirms that only one single nanobelt is assembled on the Au electrode pair.

**Figure 2:**
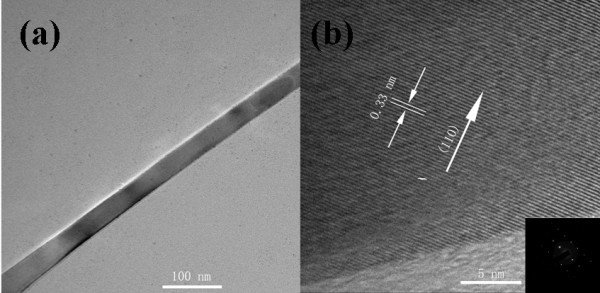
**TEM image of SnO_2 _nanobelt**. (**a**) TEM image of SnO_2 _nanobelt. (**b**) HRTEM of single SnO_2 _nanobelt. An inset image of (b) is diffraction pattern of SnO_2 _nanobelt.

Figure 2a is transmission electron microscopy (TEM) image of a single SnO_2 _nanobelt. High-resolution transmission electron microscopy (HRTEM) image of a single SnO_2 _nanobelt is obtained with a JEM-2010 transmission electron microscope (Figure 2b). It shows a single nanobelt with diameter of about 40 nm. And well-defined lattice fringe separation with 0.33 nm corresponding to (110) planes of SnO_2_. It is also in agreement with the selected area electron diffraction results (the inset of Figure 2b).

**Figure 3:**
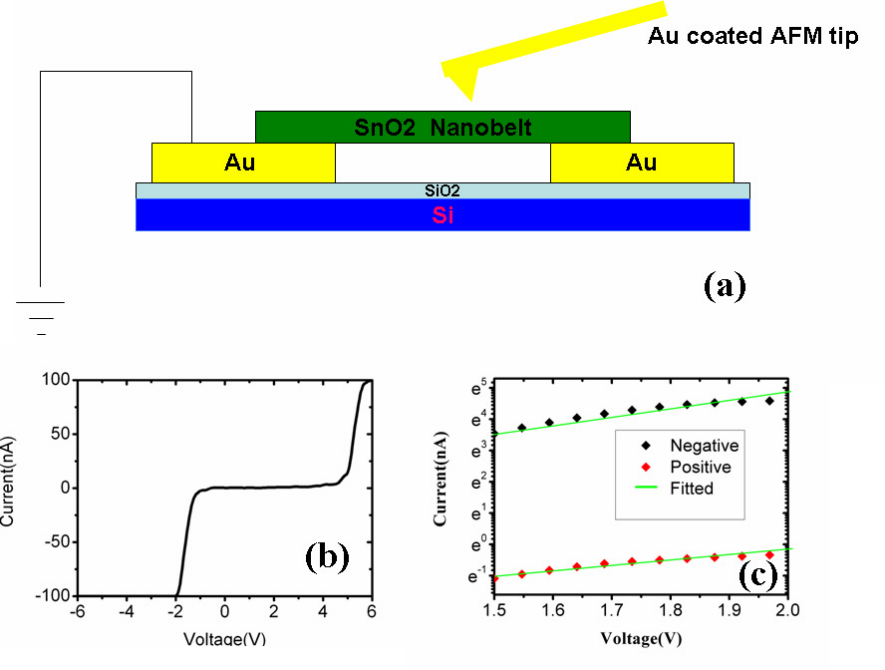
**AFM measurement**. (**a**) C-AFM measurement setup. (**b**) I-V curve measured by conductive AFM tip on the top of SnO_2 _nanobelt top surface. (**c**) Fitted lnI versus V curve at positive and negative bias voltages.

The electronic transport property of SnO_2 _nanobelt is carried out by C-AFM techniques with Au-coated silicon tip. Figure 3a is the measurement setup diagrams in our experiment. In the current-voltage (I-V) measurements, a metal/semiconductor point contact will be formed by placing the Au-coated conductive tip on SnO_2 _nanobelt top surface. And another end of the nanobelt is contact with the metal electrode, so the metal-semiconductor-metal (M-S-M) structure is formed. When a voltage bias (from +6.0 to -6.0 V) is added on the Au tip, a clearly nonlinear and asymmetric I-V curve is obtained as shown in Figure 3b.

The electronic transport properties of SnO_2 _nanowires have been studied by F. Hernandez-Ramirez in contact with Pt metal electrode [22]. The nonlinear I-V curve is attributed to the back-to-back Schottky barrier-type junctions formed between the nanowire and the metal electrode. Here, the C-AFM tip serves as one end of the electrode, and another end is the Au electrode, so the back-to-back Schottky barrier structure in series with the resistance of SnO_2 _nanobelt is formed. In this structure, the current is dominated by the reverse current of reverse-biased Schottky barrier. The I-V curves in our experiment at different bias voltage are all the reverse current of the Schottky diode. Our analysis of the reverse-biased Schottky barrier is based on the thermionic-field emission model of Padovani and Stratton [23]. Tunneling through the reverse-biased Schottky barrier becomes significant in the nano-M-S-M structure. And the tunneling current is mainly the thermionic-field emission current and the current density through the reverse-biased Schottky barrier is given by:

(1) $$\ln I=\ln\left(SJ\right)=\ln\left(SJ_{s}\right)+V\left(\frac{q}{k_{B}T}-\frac{1}{E_{0}}\right)$$

where *S *is the contact area associated with Schottky barrier, *J *is the current density through Schottky barrier, *V *is the applied revere bias, *q *is the unit charge of an electron, *K_B _*is the Boltzmann's constant, *E_0 _*is a parameter that depends on the carriers' density, *J_s _*is a slowly varying function of applied bias. Equation 1 indicates that the logarithmic of the current is linear with reverse bias. The log-scale plots of positive and negative current are shown in Figure 3c. We can see that ln*I *is linear with *V *in the intermediate bias range for both positive and negative current, which shows the typical I-V characteristic of back-to-back Schottky barriers structure. We can also find that in Eq. (1), the current value is related to contact area of the Schottky barrier. For the contact area of Au tip and SnO_2_, it is much smaller than the metal electrode due to the nanometer scale resolving of the AFM tip, which may be the main reason for the asymmetric I-V curve made on the nanobelt surface. Therefore the nonlinear and rectifying behavior in our I-V curve resulted from the Schottky contact between AFM tip/SnO_2 _nanobelt/electrode and the Schottky barriers may be related to the surface states on the nanobelt surface.

**Figure 4:**
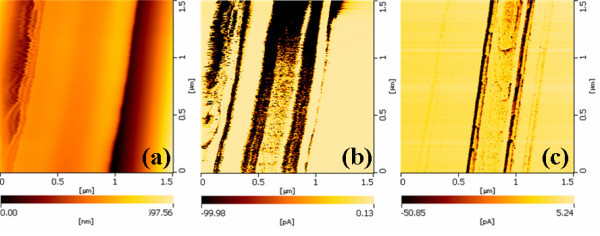
**Current images of SnO_2 _nanobelt**. (**a**) Surface topography, (**b**) current maps at -4.0 V, (c) reduced current images in the second scan at -4.0 V.

In order to illustrate and how the surface states affect the transport property of single SnO_2 _nanobelt clearly, current maps are carried out by C-AFM at negative bias voltage. The negative bias voltage here is consistent with the bias voltage at the I-V curve record on the SnO_2 _nanobelt. The voltage value (we selected -4.0 V) should be larger than 2.0 according to the I-V curves we made on the nanobelt surface. Here, current maps can provide direct transport property information on the surface of the nanobelt. It correlates the microscopy with separate device transport measurement together which can introduce us the device property of the nanobelt in nanometer scales [24]. Figure 4 is the topography and current images of single SnO_2 _nanobelt between the gaps of the metal electrode. The scan area is 1.5 × 1.5 μm, which is smaller than the gap of the electrode (5 μm). At -4.0-V bias voltages, the current maps are almost identical to that of surface geography (Figure 4a, b). In the contrast of the current images, dark and bright spots indicate relatively low and high values of the current flow through the SnO_2 _nanobelt. According to Figure 4, the current image show stripe patterns on the nanobelt surface which indicate the conducting channels of the current through the nanobelt surface. The current maps show us that the much larger current appeared at the edge and on specific area of the SnO_2 _nanobelt. From the surface geography of Figure 4a, we find that the edge of the SnO_2 _nanobelt is irregular and also there are height variations on the specific area on the surface of SnO_2 _nanobelt. And these irregular structures which are caused from the nanobelt preparation process are correlative with the stripe pattern current images of the SnO_2 _nanobelt. At the same time the surface states are formed on these defect areas due to greater number of oxygen vacancies on it.

We have demonstrated that the Schottky barriers between CuO nanowires and metallic electrodes are dominated by the surface states on the semiconductor nanowires surface, and the conductive atomic force microscopy current maps showed the current varied with the surface states of the nanowire [25]. Thus the surface states are widely existing phenomena for semiconductor nanobelts or nanowires. Here, the SnO_2 _nanobelts have some defect areas in its preparation process. So oxygen vacancies and the defect on the nanobelt would serve as surface states and impact the transport property of the nanobelt. According to the I-V curves analysis above, Schottky barrier junction is formed between the tip of AFM and the nanobelt surface. In this structure, the current is dominated by the tunneling current of reverse-biased Schottky barrier. And the tunneling current is dominated by the surface states on the nanobelt surface. That is to say, the current images of the SnO_2 _nanobelt is caused by the surface states which result from the oxygen vacancies and the defect on the nanobelt that we can analyze from the surface geography of Figure 4a. Thus the low- and high-current flow through the nanobelt will happened and can be visualized as stripe patterns in our current maps. Through the second scan of the same area under the same bias, lowered current images are obtained. The large current is decreased with repeated scan at the same area on the nanobelt surface just as Figure 4c showed. In C-AFM scans, the bias voltage is applied on the nanobelt surface, and electron is injected from the AFM tip through the depletion layer into the nanobelt. Here, the surface states tend to trap electrons further and widen the depletion layer which leads to the decrease in electron mobility as well as a decrease in the current value in the same area on the nanobelt surface. The surface states trap charge carriers in the first scan of C-AFM at -4.0 bias voltages and enhance the barrier height on the nanobelt surface, when the same bias voltage is add on the nanobelt again decreased currents image will be obtained.

The photocurrent maps are also carried out at the same bias voltage values by our photo-assisted AFM techniques with an UV light source (*λ *= 350 nm, average power of 8 mW) [14], but the current values have no obvious changes, the varied value only are several picoamperes. The surface states tend to extract electrons from SnO_2 _which can form a depletion of the charge carriers in the nanobelt. The depletion layer which is caused by the surface states can affect the conducting property of the nanobelt. Also, the depletion regions in the nanobelt can act as obstacles that hinder the electron transfer from the nanobelt to the electrode and increase the turn-on voltage of the nanobelt devices. Usually, the surface states can be modified by the exciting UV light. The UV photons can release of the trapped electrons back into the depletion region, gradually reducing the band bending. Since the nanobelts have a greater number of oxygen vacancies, the depletion region and surface field is larger, the effect of UV light is not as strong as make photocurrent changes in our current images. Surface states as the charge trapping effect in nanometer scale must be cared in nano-electronic device fabricated in the future. Thus, the electronic transport property of single nanobelt nanodevices is directly studied by correlating the microscopy with separate device measurement together. Current maps show us clearly the transport property of the nanobelt at nanometer scales.

## Conclusion

In conclusion, the electronic transport property of single SnO_2 _nanobelt was studied by conductive atomic force microscopy (C-AFM). Back-to-back Schottky barrier-type junctions were created between AFM tip/SnO_2 _nanobelt/electrode which reasoning from the I-V curve made on the nanobelt. The current images of single SnO_2 _nanobelt nanodevices were also studied by C-AFM techniques, which showed stripes patterns on the nanobelt surface. The results showed that the surface states can affect the transport property of the nanobelt device and display stripe patterns in the current images. The electronic transport property of single SnO_2 _nanodevices affect by the surface states can be important for constructing nanosensors and other nanodevices based on SnO_2 _nanobelt in nanometer scales in future.

## Competing interests

The authors declare that they have no competing interests.

## Authors' contributions

WSJ is the primary author and carried out the experiments, characterization, and acquisition of data. CG participated in analysis and interpretation of data. CK and JXH participated in language modification. ZLD is the principal investigator helped analysis and interpretation of data, drafting of the manuscript and revisions. All authors read and approved the final manuscript.
